# The role of uPAR in epithelial-mesenchymal transition in small airway epithelium of patients with chronic obstructive pulmonary disease

**DOI:** 10.1186/1465-9921-14-67

**Published:** 2013-06-28

**Authors:** Qin Wang, Yunshan Wang, Yi Zhang, Yuke Zhang, Wei Xiao

**Affiliations:** 1 Department of Respiratory Medicine, Qilu Hospital, Shandong University, Jinan, China; 2 Department of Anatomy, Shandong University School of Medicine, Jinan, China; 3 International Biotechnology Research and Development Centre, Shandong University, Weihai, China

**Keywords:** Urokinase plasminogen activator receptor, Epithelial-mesenchymal transition, Small airway epithelial cells, Chronic obstructive pulmonary disease and cigarette smoke

## Abstract

**Background:**

Epithelial-mesenchymal transition (EMT) plays a crucial role in small airway fibrosis of patients with chronic obstructive pulmonary disease (COPD). Increasing evidence suggests that the urokinase plasminogen activator receptor (uPAR) is involved in the pathogenesis of COPD. Increased uPAR expression has been implicated in the promotion of EMT in numerous cancers; however the role of uPAR in EMT in small airway epithelial cells of patients with COPD remains unclear. In this study, we investigated the degree of EMT and uPAR expression in lung epithelium of COPD patients, and verified the effect of uPAR on cigarette smoke extract (CSE)-induced EMT *in vitro*.

**Methods:**

The expression of EMT biomarkers and uPAR was assessed in lung epithelium specimens from non-smokers (n = 25), smokers (n = 25) and non-smokers with COPD (n = 10) and smokers with COPD (n = 18). The role of uPAR on CSE-induced EMT in human small airway epithelial cells (HSAEpiCs) was assessed by silencing uPAR expression *in vitro*.

**Results:**

Markers of active EMT and uPAR expression were significantly increased in the small airway epithelium of patients with COPD compared with controls. We also observed a significant correlation between uPAR and vimentin expression in the small airway epithelium. *In vitro*, CSE-induced EMT in HSAEpiCs was associated with high expression of uPAR, and targeted silencing of uPAR using shRNA inhibited CSE-induced EMT. Finally, we demonstrate that the PI3K/Akt signaling pathway is required for uPAR-mediated EMT in HSAEpiCs.

**Conclusions:**

A uPAR-dependent signaling pathway is required for CSE-induced EMT, which contributes to small airway fibrosis in COPD. We propose that increased uPAR expression in the small airway epithelium of patients with COPD participates in an active EMT process.

## Background

Chronic obstructive pulmonary disease (COPD) represents a major health problem, and is currently the fourth leading cause of death world-wide [1]. COPD typically involves two spectra of clinical or pathological presentation, chronic bronchitis and emphysema. In the majority of cases, COPD is caused by cigarette smoking, which induces chronic airway inflammation and development of emphysema, leading to irreversible airflow limitation and an accelerated decline in lung function [2]. The narrowing of small conducting airways before the onset of emphysematous destruction may explain the increased peripheral airway resistance reported in COPD [3]. These small airways (< 2 mm in internal diameter) are major sites of obstruction in patients with COPD.

Studies by Hogg et al. [4] provide the most compelling evidence that small airways (< 2 mm) undergo remodeling and fibrous thickening, based on analysis of lung tissues obtained by cancer resections or lung volume reduction surgery. The key effector cell in airway fibrogenesis is the myofibroblast. A detailed understanding of the pathways underlying myofibroblast proliferation will likely lead to the identification of important new molecular targets that may be exploited therapeutically in patients with COPD.

Airway epithelial cells may acquire a mesenchymal phenotype and serve as an important source of fibroblasts and myofibroblasts, through a process known as epithelial-mesenchymal transition (EMT) [5]. During this process, fully differentiated epithelial cells undergo phenotypic transition to fully differentiated mesenchymal cells, often fibroblasts and myofibroblasts. The interaction between EMT and airway epithelial cells has been addressed by several *in vitro* studies. Molloy et al. were the first to report an EMT process in airway epithelial cells (AECs), following the demonstration that BMP4 induces EMT in human bronchial epithelial cells [6]. McCormack and colleagues proposed that acquisition of an EMT-like phenotype by AECs is a normal aspect of wound repair. Furthermore, they suggest that diseases involving fibrosis may arise because the EMT phase of repair is prolonged by chronic injury/inflammation [7]. Recent studies have shown that cigarette smoke condensate (CSC) induces an EMT-like process in human bronchial epithelial cells (BEAS-2B) [8], however the effect of cigarette smoke on EMT of small airway epithelial cells is currently poorly understood. Sohal et al. provide evidence that EMT is indeed an active process in the airways of smokers, particularly in those of current-smoking COPD patients. This was based on immunohistochemical analysis of epithelial and mesenchymal markers in the basal layer of the epithelium and in the fragmented reticular basement membrane (Rbm) [9]. Active airway EMT in COPD patients may be related to subsequent fibrotic activity in the sub-epithelial tissue, however the mechanisms involved in this process remain to be elucidated.

Our group previously demonstrated that urokinase plasminogen activator receptor (uPAR) is highly expressed in the small airway epithelia of patients with COPD compared with normal controls [10]. Furthermore, uPAR expression was also related to clinical parameters of airflow limitation. The uPAR protein is a modulator of the plasminogen pathway, which cleaves and activates urokinase-type plasminogen activator (uPA) [11]. uPAR-uPA is involved in the proteolytic activation of plasminogen to plasmin, which in turn degrades fibrin and other extracellular matrix (ECM) components and activates matrix metalloproteases. Given the known biology of uPAR, this receptor may be implicated in small airway remodeling in COPD, leading to the eventual decline in lung function. In support of this, studies by Lester et al. [12] demonstrated that uPAR activates diverse cell signaling pathways, including ERK/MAPK, Rac1, Akt and glycogen synthase kinase-3β (GSK-3β), that cooperatively induce EMT in hypoxic breast cancer cells.

Although the role of uPAR in small airway disease has previously been evaluated in COPD, the role of uPAR in active EMT in the small airways of COPD patients remains unknown. In this study, we characterized mesenchymal marker expression in small airway epithelial cells of COPD patients. In addition, because cigarette smoke exposure is associated with COPD pathogenesis, we investigated cigarette smoke extract (CSE)-induced EMT in human small airway epithelial cells and examined the effects of uPAR on this process *in vitro*. Our study indicates that uPAR expression is related to EMT in human small airway epithelium, demonstrating a critical role for uPAR in COPD pathogenesis.

## Methods

### Patients

Lung tissues were obtained from 78 patients (25 non-smokers, 25 smokers without COPD, 10 non-smokers with COPD and 18 smokers with COPD) at Qilu Hospital (Jinan, China) following lobectomy or pneumonectomy for various medical reasons. The diagnosis of COPD was made according to the guidelines of the Global Initiative for Chronic Obstructive Lung Disease [13]. No subjects received corticosteroids (oral or inhaled) prior to tissue collection. All experiments were approved by the ethics committee of Qilu Hospital and informed consent was obtained from all patients prior to specimen collection.

### Immunohistochemistry

Serial sections (4 μM) of formalin-fixed, paraffin-embedded lung tissue were used for immunohistochemical analysis. Sections were immunostained with primary antibodies recognizing E-cadherin (24E10, Cell Signaling Technology, Beverly, MA, USA), vimentin (D21H3, Cell Signaling Technology) and uPAR (10G7, Santa Cruz Biotechnology, Santa Cruz, CA, USA) and visualized using the avidin-biotin-peroxidase (ABC) complex (ZhongShan Biotech, Beijing, China) method. Color development was performed using a DAB color development kit (ZhongShan Biotech). Images were captured using an OLYMPUS IX81 light microscope (Olympus, Tokyo, Japan) fitted with a SPOT camera. Image analysis was performed using Image-Pro Plus 6.0 software (Media Cybernetics, Silver Spring, MD, USA). All slides were analyzed in a single batch by a single experienced observer with quality assurance on randomly selected slides provided by a professional academic pathologist.

### Preparation of CSE

CSE was prepared using a modified method described by Aoshiba [14]. Briefly, one filterless commercial cigarette (13 mg of tar and 1.2 mg of nicotine per cigarette) was combusted using a modified, syringe-driven apparatus. Mainstream smoke was bubbled through 20 ml of serum-free Ham‘s F12 nutrient medium (F12). The resulting suspension was adjusted to pH 7.4 with concentrated NaOH and filtered through a 0.22-μm pore filter to remove bacteria and large particles. This solution (designated as a 100% CSE solution) was used within 30 min of preparation.

### Cell culture

Human small airway epithelial cells (HSAEpiCs) were obtained from ScienCell Research Laboratories (Cat. No. 3231). Cells were cultured in Small Airway Epithelial Cell Medium (SAEpiCM) at 37°C in a water-saturated atmosphere with 5% carbon dioxide.

### uPAR-specific short hairpin RNA inhibition

For knockdown of uPAR expression in HSAEpiCs, short hairpin RNAs (shRNAs) targeting human uPAR (shuPAR1, GCCGTTACCTCGAATGCAT and shuPAR2, GGUGAAGAAGGGCGUCCAA) were cloned into the pSuper vector as previously described [15]. Control oligonucleotides corresponding to the inverse uPAR shRNA sequences were also prepared. Sub-confluent (75%) cell monolayers were transfected with pSuper-shRNA targeting uPAR or empty vector using Lipofectamine 2000 (Invitrogen, Carlsbad, CA, USA), in accordance with the manufacturer’s instructions. After 24 h, cells were trypsinized and subjected to various experiments. uPAR knockdown was assessed by real-time-polymerase chain reaction (PCR) and western blotting.

### Real-time PCR

Total RNA was extracted using Trizol reagent (Invitrogen) according to the manufacturer‘s instructions. First strand cDNA was synthesized using Superscript II reverse transcriptase (Invitrogen). Real-time PCR reactions were prepared using SYBR® Green Real time PCR Master Mix (Invitrogen) and PCR was performed with an ABI PRISM 7900 HT Sequence Detection System. Primers used for the amplification of indicated genes are listed in Additional file 1: Table S1. Endogenous *GAPDH* was used as a normalization control. Relative quantification of mRNA was performed using the comparative CT method [16].

### Western blot analysis

Cells extracts were prepared using ice-cold RIPA buffer (20 mM sodium phosphate, 150 mM NaCl, pH 7.4, 1% NP-40, 0.1% SDS and 0.5% deoxycholic acid) containing complete protease inhibitor cocktail (Roche) and 1 mM sodium orthovanadate. Proteins were separated by 10% SDS-PAGE and transferred to PVDF membranes and probed with antibodies detecting human uPAR, E-cadherin, α-catenin (610194, BD Transduction Laboratories), N-cadherin (610920, BD Transduction Laboratories), α-SMA (324316, Santa Cruz), phospho-Akt (#4060, Cell Signaling Technology), Akt (#4691, Cell Signaling Technology), Snail (10433, Santa Cruz), phospho-GSK-3β (81494, Santa Cruz) and GSK-3β (53931, Santa Cruz). β-actin (#4970, Cell Signaling Technology) was used as an endogenous loading control.

### Statistical analysis

Patient’s age, clinical score and real time PCR expression data are expressed as the mean ± standard deviation. The Kruskal–Wallis and Mann–Whitney U-test were used for comparisons between patient groups. The Spearman test was used for correlation analyses. Student’s t test was used for *in vitro* experiments on HSAEpiCs. P values < 0.05 were accepted as statistically significant.

## Results

### Patient demographic characteristics

Demographic characteristics and functional evaluation of the patient study groups are shown in Table 1. All patient groups exhibited a similar age range. The forced expiratory volume (FEV_1_%) of predicted and the FEV_1_/forced vital capacity (FVC) ratio were significantly lower in patients with COPD compared with control subjects (*P* < 0.01). In contrast, we observed no difference in FEV_1_% of predicted or in the FEV_1_/FVC ratio between smokers with COPD and non-smokers with COPD (*P* > 0.05).

**Table 1:** Demographic characteristics of the subjects

	**Non-smokers(NC) n = 25**	**Smokers (NS) n = 25**	**Non-smokers with COPD (CN) n = 10**	**Smokers with COPD (CS) n = 18**
Sex (female/male)	20/5	1/24	9/1	0/18
Age (years)	55 ± 9	56 ± 11	59 ± 7	61 ± 9
Smoking history, pack-years	**―**	26 ± 14	―	37 ± 25
FEV1,% predicted	97 ± 15	99 ± 11	65 ± 17	70 ± 16
FEV1/FVC%	84 ± 8	80 ± 7	56 ± 7	61 ± 5
GOLD stage				
1	―	―	1	1
2	―	―	7	12
3	―	―	2	5
4	―	―	―	―

Values are given as mean ± SD.

Pack-year = 1 year smoking 20 cigarettes per day.

COPD, chronic obstructive pulmonary disease; FEV1, forced expiratory volume in 1 s; FVC, forced vital capacity.

### EMT in patient small airway epithelium cells

**Figure 1:**
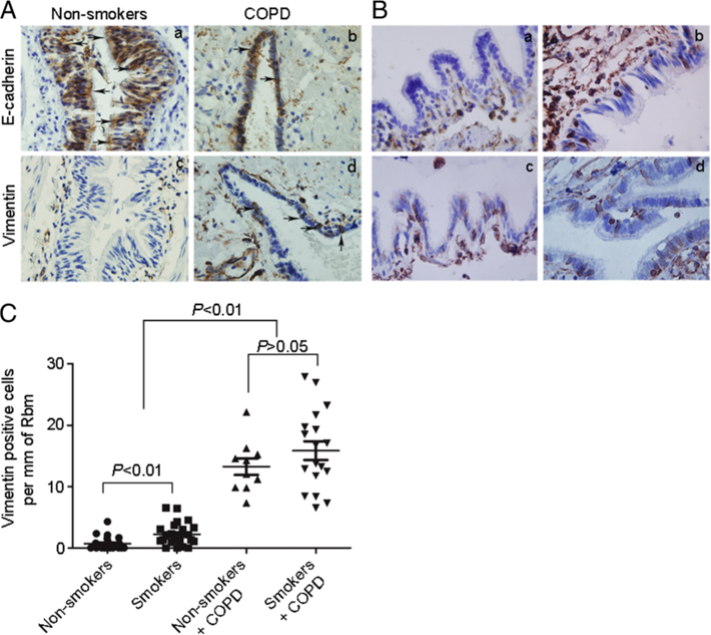
**Immunohistochemistry for EMT biomarkers in small airways epithelium of patients with chronic obstructive pulmonary disease (COPD).** Small airways sections from non-smokers (n = 25), smokers (n = 25), non-smokers with COPD (n = 10), and smokers with COPD (n = 18) were immunostained. (**A**) Epithelial marker E-cadherin (brown) and mesenchymal marker vimentin (brown) staining are observed in small airways epithelium from a non-smoker and a patient with COPD with serial sections. Black arrows show the main distribution of the positive immunostaining regions. (**B**) Mesenchymal marker vimentin (brown) from non-smokers (a), smokers (b), non-smokers with COPD (c) and smokers with COPD (d). (**C**) Quantification of mesenchymal marker vimentin positive cells in the small airways epithelium. The number of positive epithelial cells per mm reticular basement membrane (Rbm) in small airways. *P* values in figure were obtained by Mann–Whitney U test analyses. Images were obtained using a 100 × oil-immersion objective.

Thickening of the walls of small airways is caused by fibrosis and infiltration of inflammatory cells [17]. Because EMT is associated with fibrosis, we first assessed the expression of EMT biomarkers in distal airway epithelial cells from patients with lung resection. Expression of the epithelial marker, E-cadherin and the mesenchymal marker, vimentin was assessed in small airway epithelium from a non-smoker and a patient with COPD by immunohistochemistry (Figure 1A). We observed significant immunostaining of E-cadherin in small airway epithelial cells from a non-smoker, while vimentin expression was absent. In contrast, E-cadherin was expressed at lower levels in the small airway epithelium of a patient with COPD, while vimentin was detected in the distal airway epithelium. A marked increase in the number of vimentin positive cells was observed within the small airway epithelium of smokers with COPD, non-smokers with COPD and smokers compared with non-smokers without COPD (Figure 1B), especially in patients with COPD. In contrast, we observed no significant difference in the number of vimentin positive cells between smokers with COPD and non-smokers with COPD (*P* =0.4493; Figure 1C).

### uPAR expression in human small airway epithelium

**Figure 2:**
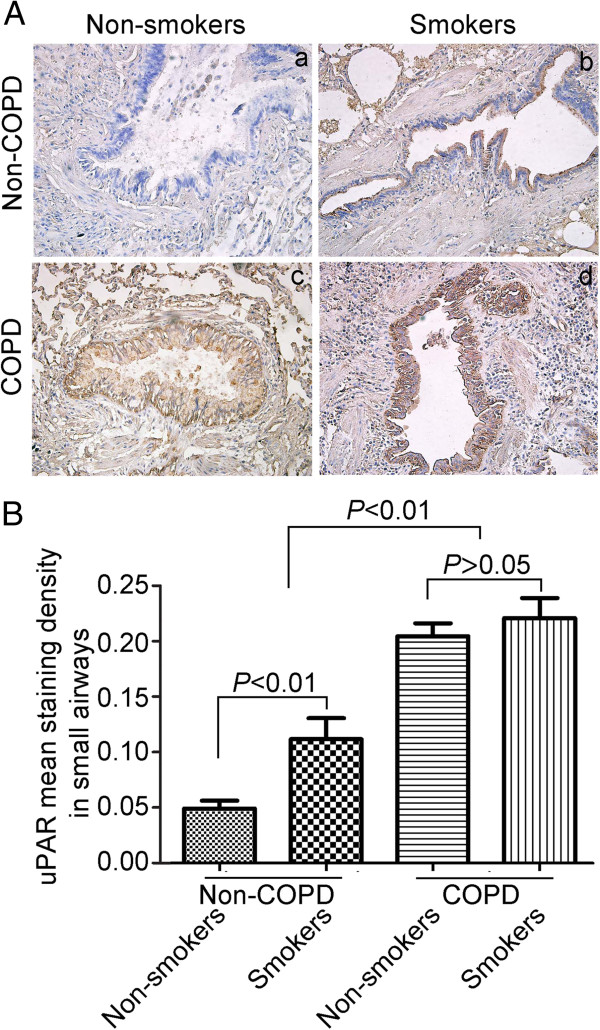
**Urokinase plasminogen activator receptor (uPAR) immunostaining in small airways epithelium of patients with COPD.** (**A**) uPAR staining in the epithelium of small airways from non-smokers (a), smokers (b), non-smokers with COPD (c) and smokers with COPD (d) (original × 200). (**B**) Quantification of uPAR protein levels in the small airways epithelium. The mean staining density in small airways.

To investigate whether EMT in the small airways of COPD may be explained by an increase in uPAR, we next investigated uPAR expression in human lung tissues. Our previous study confirmed that uPAR expression was significantly elevated in the small airway epithelia of subjects with COPD (n = 16) compared with control subjects [10]. In this study, we assessed uPAR expression by immunostaining in small airway epithelial cells in a larger patient cohort (Figure 2A). The expression of uPAR in small airway epithelial cells was assessed using the mean staining density calculated using Image-Pro Plus 6.0. uPAR expression was increased in the epithelium of distal airways from smokers and patients with COPD, compared with non-smokers. Moreover, we observed a significant increase in uPAR levels in the epithelium of patients with COPD compared with non-smokers and smokers (*P* < 0.01; Figure 2B). There is no significant difference in uPAR levels between smokers with COPD and non-smokers with COPD (*P* > 0.05; Figure 2B).

### Correlation analysis

**Figure 3:**
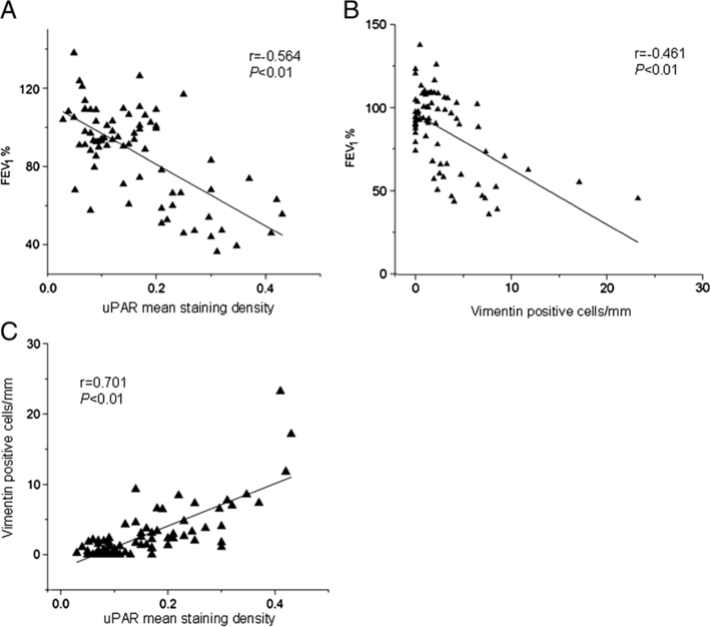
**Correlations between the expression of uPAR or mesenchymal marker vimentin positive cells in small airways and functional parameters.** (**A**) The expression of uPAR in small airways of 78 subjects was correlated with FEV_1_% of predicted, as an index of airflow obstruction severity in COPD. (**B**) Mesenchymal marker vimentin positive cells per mm in small airways was correlated with FEV_1_% of predicted. (**C**) The expression of uPAR in small airways was correlated with vimentin positive cells per mm.

We next investigated whether uPAR or vimentin expression in the small airways correlated with FEV_1_% of predicted. The severity of postbronchodilator FEV_1_% of predicted is recommended for the diagnosis and assessment of COPD severity according to the Global Initiative for Chronic Obstructive Lung Disease (GOLD). We also evaluated the relationship between uPAR and vimentin expression in the epithelium of distal airways. We observed a significant inverse correlation between FEV_1_% and uPAR expression (r = −0.564, *P* < 0.01, Figure 3A) and vimentin expression (r = −0.461, *P* < 0.01, Figure 3B). A significant correlation between the mean staining density of uPAR and the number of vimentin positive cells (per mm basal membrane) in the small airway epithelium was also observed (r =0.701, *P* < 0.01, Figure 3C).

### CSE-induced EMT in cultured HSAEpiCs

**Figure 4:**
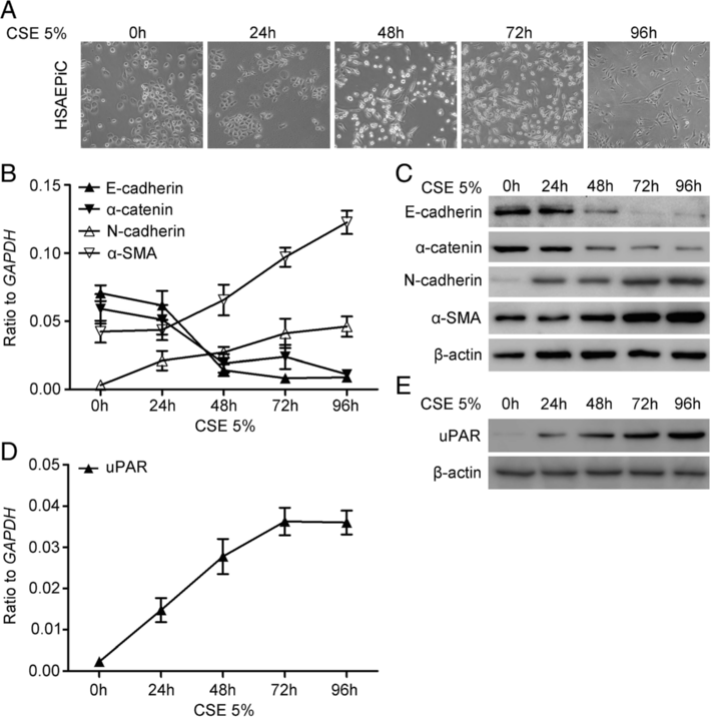
**Cigarette smoke extract (CSE) induces epithelial-mesenchymal transition and increases uPAR levels in cultured human small airway epithelial cells (HSAEpiC).** (**A**) Cells were cultured for variable time points with 5% CSE. Cell images were captured by phase-contrast microscopy. (**B**) Epithelial markers *E-cadherin* and α*-catenin*, mesenchymal markers *N-cadherin* and α*-smooth muscle actin* (α*-SMA*) mRNA levels were determined by Real-time PCR (mean ± SEM; n = 3). (**C**) Epithelial markers E-cadherin and α-catenin, mesenchymal markers N-cadherin and α-SMA protein levels were determined by Western blot. The original extracts were subjected to Western blot analysis for β-actin, as a loading control. (**D**) *UPAR* mRNA level was determined by Real-time PCR (mean ± SEM; n = 3). (**E**) uPAR protein level was determined by Western blot.

Cigarette smoking is the most commonly encountered risk factor for COPD. To better model this microenvironment, we cultured HSAEpiCs in the presence of CSE. Following treatment with 5% CSE for 48 h, HSAEpiCs, which typically appear epithelial with well-developed cell junctions, acquired a spindle shape and exhibited a general loss of cell contact (Figure 4A).

To examine whether CSE induces EMT in HSAEpiCs, cells were exposed to 5% CSE for various time points. The expression of E-cadherin and α-catenin epithelial markers was significantly decreased in HSAEpiCs at both mRNA and protein levels in response to 5% CSE, in a time-dependent fashion. In contrast, CSE increased the expression of N-cadherin and α-smooth muscle actin (α-SMA) mesenchymal markers in HSAEpiCs in a time-dependent manner (Figure 4B, C).

### CSE-induced uPAR signaling pathway activation in cultured HSAEpiCs

To further investigate the role of cigarette smoke exposure in EMT, we evaluated the expression of uPAR in CSE stimulated HSAEpiCs at both mRNA and protein levels. Expression of *UPAR* mRNA was markedly increased in a time-dependent manner following CSE exposure, reaching peak levels after 72 h (Figure 4D). Treatment of cells with CSE also led to a significant increase in uPAR protein expression in a time-dependent manner (Figure 4E).

### uPAR is required for CSE-induced EMT in HSAEpiCs

**Figure 5:**
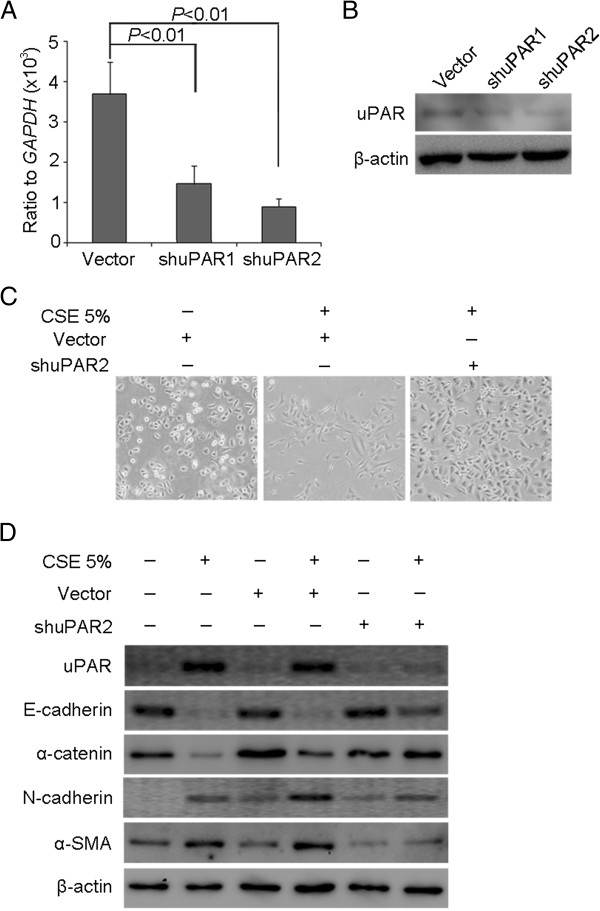
**uPAR is required for CSE-induced EMT in HSAEpiC cells.** (**A**) Cells expressing empty vector, shuPAR1 cells, and shuPAR2 cells were cultured for 24 h. *UPAR* mRNA was determined by Real-time PCR (mean ± SEM; n = 3). (**B**) uPAR protein level was determined by Western blot. (**C**) Empty vector and shuPAR2 cells were cultured for 72 h with 5% CSE. Cell images were captured by phase-contrast microscopy. (**D**) Parental cells, empty vector and shuPAR2 cells were cultured for 72 h without or with 5% CSE. Cell extracts were subjected to Western blot analysis for epithelial markers E-cadherin and α-catenin, mesenchymal markers N-cadherin and α-SMA. β-actin was used as a loading control.

To further investigate the requirement for uPAR in CSE-induced EMT, we performed targeted knockdown of uPAR in HSAEpiCs using the shRNA silencing vector, pSuper-shuPAR. We established two knockdown clones (shuPAR1 and shuPAR2) expressing small and negligible levels of uPAR protein. Inhibition of uPAR expression in individual clones compared with empty vector transduced cells was confirmed by real-time PCR and western blot (*P* < 0.01, Figure 5A, B). Analysis of morphological changes following treatment of cells with 5% CSE for 72 h revealed that uPAR knockdown (shuPAR2) caused a decrease in the population of spindle shaped cells (Figure 5C).

To test whether uPAR is responsible for CSE-induced EMT, we examined the expression of several epithelial and mesenchymal marker proteins by western blotting. CSE-treated control vector HSAEpiCs exhibited loss of cell surface E-cadherin and α-catenin, whereas N-cadherin and α-SMA were clearly detected, in keeping with the changes observed with parental cells. In contrast, CSE-treated shuPAR2 cells exhibited increased E-cadherin and α-catenin and decreased N-cadherin and α-SMA expression (Figure 5D).

### PI3K/Akt signaling pathway is required for uPAR-mediated EMT in HSAEpiCs

**Figure 6:**
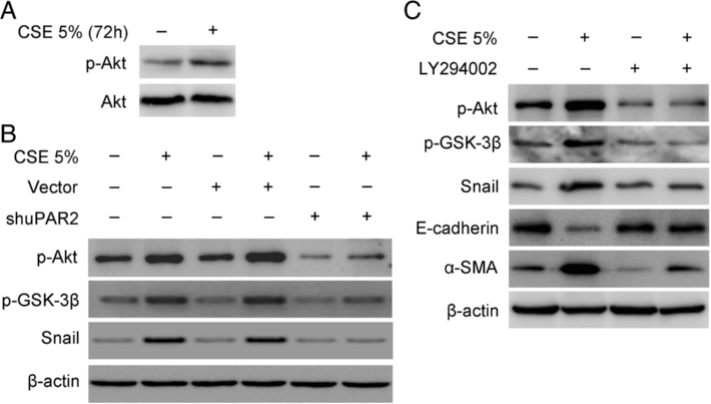
**PI3K/Akt signaling pathway is required for uPAR-mediated EMT in HSAEpiC Cells.** (**A**) Cells were cultured for 72 h without or with 5% CSE. Cell extracts were subjected to Western blot to detect phosphorylated Akt and total Akt. (**B**) Parental cells, empty vector and shuPAR2 cells were cultured for 72 h without or with 5% CSE. Cell extracts were subjected to Western blot analysis for phosphorylated Akt (p-Akt), phosphorylated GSK-3β (p-GSK-3β), Snail, and β-actin. (**C**) Cells were treated with 10 μM of the PI3K inhibitor LY294002 or with vehicle (control) for 72 h without or with 5% CSE. Cell extracts were subjected to Western blot analysis to detect p-Akt, p-GSK-3β, Snail, E-cadherin, α-SMA and β-actin (representative of three studies).

We hypothesized that activation of uPAR-dependent cell signaling may be responsible for the molecular and morphological changes observed in HSAEpiCs in response to CSE treatment. Recent studies indicate that the PI3K-Akt pathway is activated by uPA binding to uPAR [18,19]. Indeed, inhibition of PI3K/Akt signaling using the PI3K/Akt inhibitor, LY294002, impaired uPAR morphogenic activity [20-22]. To test this hypothesis, we examined base-line levels of Akt activation in HSAEpiCs cultured in 5% CSE for 72 h by western blot. We observed a significant increase in phosphorylated Akt (p-Akt) in CSE-treated HSAEpiCs (Figure 6A).

To determine whether uPAR is necessary for Akt activation in CSE-treated HSAEpiCs, we tested the effect of uPAR gene silencing on Akt activation. Western blot analysis revealed activation of PI3K-dependent Akt phosphorylation in parental cells and cells transduced with empty vector following treatment with CSE. In contrast, the level of phosphorylated Akt was substantially inhibited in shuPAR2 cells cultured with CSE compared with controls (Figure 6B).

During EMT, Akt-induced GSK-3β phosphorylation leads to GSK-3β inactivation. GSK-3β regulates Snail activity by inhibiting Snail expression, promoting Snail degradation and decreasing nuclear localization. Control cells treated with 5% CSE exhibited EMT at 72 h coinciding with upregulation of GSK-3β phosphorylation and Snail protein levels. Treatment of cells with 10 μM LY294002 blocked the induction of GSK-3β phosphorylation, Snail and α-SMA (Figure 6C). LY294002 also preserved expression of the epithelial cell marker E-cadherin in HSAEpiCs treated with CSE. These results suggest that uPAR-dependent Akt activation is essential for CSE-induced EMT.

## Discussion

In this study, we demonstrate for the first time that uPAR is a regulator involved in the EMT process in the small airways of patients with COPD. Our results demonstrate a significant increase in uPAR expression levels and in the degree of active EMT in the small airway epithelium of patients with COPD compared with non-smokers and smokers with normal lung function. Furthermore, we observed a significant correlation between uPAR expression and EMT in the small airway epithelium of COPD patients. CSE-induced EMT in cultured HSAEpiCs was accompanied by significant induction of uPAR expression, and targeted silencing of uPAR by shRNA inhibited CSE-induced EMT. Taken together, these data suggest that uPAR plays a critical role in CSE-induced EMT. In addition, we demonstrate that the PI3K/AKT signaling pathway is required for uPAR-mediated EMT in HSAEpiCs. Collectively, these results demonstrate that uPAR participates in the EMT process in the small airway epithelium of patients with COPD.

COPD is characterized by airflow limitation that is not completely reversible. Reduced FEV1 characterizes airflow limitation in COPD, and likely occurs because of small airway disease [23]. Hogg and colleagues showed that obstruction of the small airways in COPD is associated with thickening of the airway wall via remodeling processes related to tissue repair [24]. Fibrosis of the small airways is caused by impaired repair following injury to the bronchiolar epithelium [25]. To date, however, the exact mechanisms underlying small airway remodeling in COPD remain poorly understood. EMT is associated with tissue regeneration and fibrosis. EMT refers to a series of phenotypic and molecular changes that occur during various steps of embryonic development, but also in the development of fibrosis and cancer progression. Epithelial cells, via EMTs, are as important precursors of the fibroblasts and myofibroblasts that arise during the course of fibrosis [26]. Although EMT in the airways is implicated in COPD pathogenesis, the mechanisms leading to EMT in the small airways of patients with COPD remain poorly unclear.

Sohal et al. recently observed potential activation of an EMT program in large airways taken from endobronchial biopsies of smokers and patients with COPD [27]. It should be noted, however, that airway remodeling in COPD is mainly located in small conducting airways, and that large and small airways differ in their anatomical and pathophysiological characteristics. Taking this into account, Milara et al. used primary human bronchial epithelial cells (HBECs) from small human bronchi of nonsmokers, smokers and patients with COPD. They showed that the EMT process was present in bronchial epithelial cells of the small bronchi of smokers and patients with COPD and was activated by cigarette smoke *in vitro*[28]. Although this study demonstrated that cigarette smoke may induce EMT by modulating the TGF-β1 pathway as well as ROS and cAMP levels in primary HBECs, the molecular mechanisms of EMT in the epithelial cells of small airways in COPD patients are still unclear. Previously, Wang et al. identified uPAR expression as an important factor in the progression of COPD by pathway analysis of a signature set of 203 differentially regulated genes [29]. We also showed that uPAR, which can promote EMT in several tumor cell systems [30-32], is highly expressed in the small airway epithelium of patients with COPD compared with controls [10]. However, to date, the role of active EMT in small airways of patients with COPD remains unknown.

We first assessed the expression of EMT biomarkers by immunostaining in small airway epithelial cells from patients with lung resection. We observed significant expression of the epithelial marker, E-cadherin, in the small airway epithelium of non-smokers while vimentin expression was nearly absent. In contrast, E-cadherin was expressed at lower levels in the small airway epithelium of smokers and patients with COPD; however vimentin was clearly observed within the distal airway epithelium. We also observed a marked increase in the number of vimentin positive staining cells within the small airway epithelium of patients with COPD; however no difference was seen in the number of vimentin positive cells between smokers with COPD and nonsmokers with COPD. From the above results, we conclude that cigarette smoke is not the only major factor promoting active EMT, but that other risk factors contribute to airway EMT in COPD. Subsequently, we assessed uPAR expression in small airway epithelial cells in a large patient cohort. uPAR expression was increased in the epithelium of distal airways from smokers and patients with COPD compared with nonsmokers, especially in patients with COPD, in keeping with our previous study [10]. Furthermore, we observed a significant inverse correlation between FEV_1_% and uPAR or vimentin expression. A significant correlation was also observed between the uPAR expression and vimentin positive cells (per mm of basal membrane) in the small airway epithelium. These results indicate that increased uPAR levels may promote EMT in small airways, thus accounting for the previously observed reduced capacity in COPD patients.

Cigarette smoke is widely used in *in vitro* studies because of its relevance in the pathogenesis of COPD. We also used this model to investigate mechanisms underlying EMT in COPD. Treatment of HSAEpiCs with CSE led to the acquisition of a fibroblast-like morphology and general loss of cell contacts. This was accompanied by the downregulation of epithelial markers such as E-cadherin and α-catenin, and simultaneous upregulation of mesenchymal markers such as N-cadherin and α-SMA. The levels of uPAR mRNA and protein were markedly increased after exposure of HSAEpiCs to CSE. To test whether uPAR is responsible for CSE-induced EMT, we suppressed uPAR expression using shRNA. This led to inhibition of CSE-induced EMT in HSAEpiCs, indicating that uPAR is required for CSE-induced EMT in HSAEpiC.

Several cell signaling factors have been implicated in this active EMT process. PI3K/Akt is known to promote EMT by regulating the activity of GSK3β, which targets Snail for degradation by suppressing NF-κB-dependent Snail expression [33,34]. The activities of GSK3β limit the ability of Snail to function as an E-cadherin transcriptional repressor [35]. Other oncogenes, including Rac1, c-Src and Ras have also been implicated in the EMT process [36-38]. Because PI3K/Akt, Rac1, c-Src and Ras are all activated downstream of uPAR [39], we therefore favor a model in which CSE-induced uPAR expression activates cell signaling via diverse pathways that are complementary in inducing the full spectrum of cellular changes observed in EMT.

To test this model, we studied a uPAR-dependent cell signaling pathway in HSAEpiCs. We showed that PI3K/Akt is activated in HSAEpiCs following treatment with CSE, and this response was blocked by silencing uPAR. Treatment of cells with the PI3K inhibitor, LY294002, also inhibited CSE-induced GSK3β phosphorylation and Snail expression and preserved cell surface E-cadherin. Our finding that uPAR regulates PI3K/Akt activity, and consequently inhibits GSK3β activity, suggests that the uPAR pathway may also contribute to various functions/phenotypes modulated by PI3K/Akt and GSK3β. In our study, the effect of uPAR on EMT via regulation of a PI3K/Akt/GSK3β signaling module is very significant. The link between uPAR and Akt activation by CSE provides one mechanism by which uPAR may ultimately regulate Snail expression and thus promote EMT.

## Conclusions

In summary, we demonstrated that uPAR is overexpressed in the distal airways of COPD patients, and this correlates with the degree of airflow obstruction and EMT activation of small airway epithelial cells. We also demonstrated that CSE induces diverse molecular and phenotypic changes in HSAEpiCs that are consistent with EMT. Furthermore, we showed that cell signaling factors previously shown to be involved in EMT, including PI3K/Akt, GSK-3β and Snail, are activated in CSE-induced HSAEpiCs. These EMT-associated molecular and phenotypic changes are due to CSE-induced uPAR expression and activation of uPAR dependent cell signaling. We conclude that EMT may be induced in the small airway epithelium of patients with COPD, via a mechanism that involves activation of uPAR-dependent cell signaling.

## Competing interests

The authors declare that they have no competing interests.

## Authors’ contributions

QW: performed the histological, cells experiment, statistical analysis, conceiving and designing the study, and writing of paper.YSW: participated in the study in vitro and revising the manuscript.YZ : advised on histology strategy and quality control.YKZ: performed the tissue processing.WX: conceived the study, participated in designing the study and revised the manuscript. All authors read and approved the final manuscript.

## Supplementary Material

Additional file 1: Table S1. Primer sequences used for qRT-PCR. Click here for file
