# Supplementary material for: The role of uPAR in epithelial-mesenchymal transition in small airway epithelium of patients with chronic obstructive pulmonary disease
Source: Respir Res. 2013 Jun 28;14(1):67. doi: 10.1186/1465-9921-14-67 (PMC3700841; doi:10.1186/1465-9921-14-67)
Supplement: Additional file 1: Table S1. — Primer sequences used for qRT-PCR. [file 1465-9921-14-67-S1.docx]

Supplemental Table S1. Primer sequences used for qRT-PCR

| Primer | Sequence(5’ to 3’) |
| --- | --- |
| GAPDH - Forward | GCTGGCGCTGAGTACGTCGT |
| GAPDH - Reverse ward | ACGTTGGCAGTGGGGACACG |
| uPAR - Forward | CTGGAGCTTGAAAATCTGCCG |
| uPAR - Reverse ward | GGTTTTTCGGTTCGTGAGTGC |
| α-catenin - Forward | TCATTGTGGACCCCTTGAGC |
| α-catenin - Reverse ward | TTACGTCCAGCATTGCCCAT |
| α-SMA - Forward | CCCTTGAGAAGAGTTACGAGTTG |
| α-SMA - Reverse ward | ATGATGCTGTTGTAGGTGGTTTC |
| E-cadherin - Forward | TGGGCTGGACCGAGAGAGTTTC |
| E-cadherin - Reverse ward | ATCCAGCACATCCACGGTGACG |
| N-cadherin - Forward | CCGGTTTCATTTGAGGGCACATGC |
| N-cadherin - Reverse ward | GCCGTGGCTGTGTTTGAAAGGC |
